# Laboratory-Acquired Dengue Virus Infection—A Case Report

**DOI:** 10.1371/journal.pntd.0001324

**Published:** 2011-11-29

**Authors:** Sumudu Britton, Andrew F. van den Hurk, Russell J. Simmons, Alyssa T. Pyke, Judith A. Northill, James McCarthy, Joe McCormack

**Affiliations:** 1 Department of Infectious Diseases, Mater Health Services, Brisbane, Australia; 2 Public Health Virology, Queensland Health Forensic and Scientific Services, Brisbane, Australia; 3 Queensland Institute of Medical Research, Brisbane, Australia; 4 University of Queensland, Brisbane, Australia; Tropical Medicine Institute Pedro Kourí, Cuba

## Introduction

The WHO estimates there may be 50 million dengue virus (DENV) infections worldwide every year, with the disease being endemic in more than 100 countries [1]. There has been a dramatic rise in the incidence of dengue in recent decades, making this an arbovirus of major international public health concern. Dengue viruses belong to the family *Flaviviridae* and are transmitted between humans via infected female *Aedes* mosquitoes, particularly *Aedes aegypti*. In the state of Queensland, Australia, infected travellers from overseas have facilitated numerous DENV outbreaks [2], [3]. However, these outbreaks are limited to the far north of the state, the only area of Australia where *Ae. aegypti* occurs [4].

There have been case reports of non-vector, healthcare-associated transmission of DENVs—four cases of percutaneous transmission via needlestick injuries, mucocutaneous transmission through a blood splash to the face, vertical transmission, and transmission via bone marrow transplant (summarised in [5]). We report the first case to our knowledge of DENV infection acquired by a laboratory scientist conducting mosquito infection and transmission experiments.

## The Case

The patient, a scientist at a research laboratory, was referred to a public hospital emergency department by a general practitioner after presenting with fever, myalgia, and a rash. The patient resided in an area of Australia where *Ae.aegypti* has not been reported since the mid-1950 s [4]. The patient had travelled to Argentina 4 weeks earlier but did not have recent contact with similarly unwell persons or pets and had no other medical history of clinical significance. Ten days prior to hospital admission, the patient had performed a routine laboratory experiment involving the primary infection of colony mosquitoes with DENV-type 2 (DENV-2) via an artificial membrane feeding apparatus. During the procedure the patient had worn personal protective equipment commensurate with what is required for working with DENV in Australian laboratories, including gown, gloves, and eye protection [6]. The patient reported a bite from an escaped non-bloodfed mosquito during that day but denied needlestick injury or mucocutaneous contact with the blood/virus mixture. Four days later, the patient developed high fever associated with marked lethargy and fatigue, which progressed to myalgias and severe back pain over the subsequent 48 hours. Three days after the onset of fever, a fine, macular, blanching rash developed that was generalised and pruritic. Later findings following hospital admission demonstrated evidence of neutropenia (neutrophil count 0.7×10^9^/L) and thrombocytopenia (platelet count 79×10^9^/L). Results of liver function tests also revealed elevated levels of alanine aminotransferase (578 U/L) and aspartate aminotransferase (630 U/L). Ten days following the onset of fever, DENV infection was confirmed by detection of specific DENV-2 nucleic acid by real-time TaqMan reverse transcriptase polymerase chain reaction (G. Smith, unpublished data) and anti-DENV-2 IgM antibodies [7] in the patient's serum. Subsequent testing of a convalescent phase sample collected 17 days after the first specimen further demonstrated the presence of anti-DENV IgM antibodies. Of note, seroconversion of anti-flavivirus IgG antibodies was also detected, suggesting that this was an acute infection.

**Figure 1:**
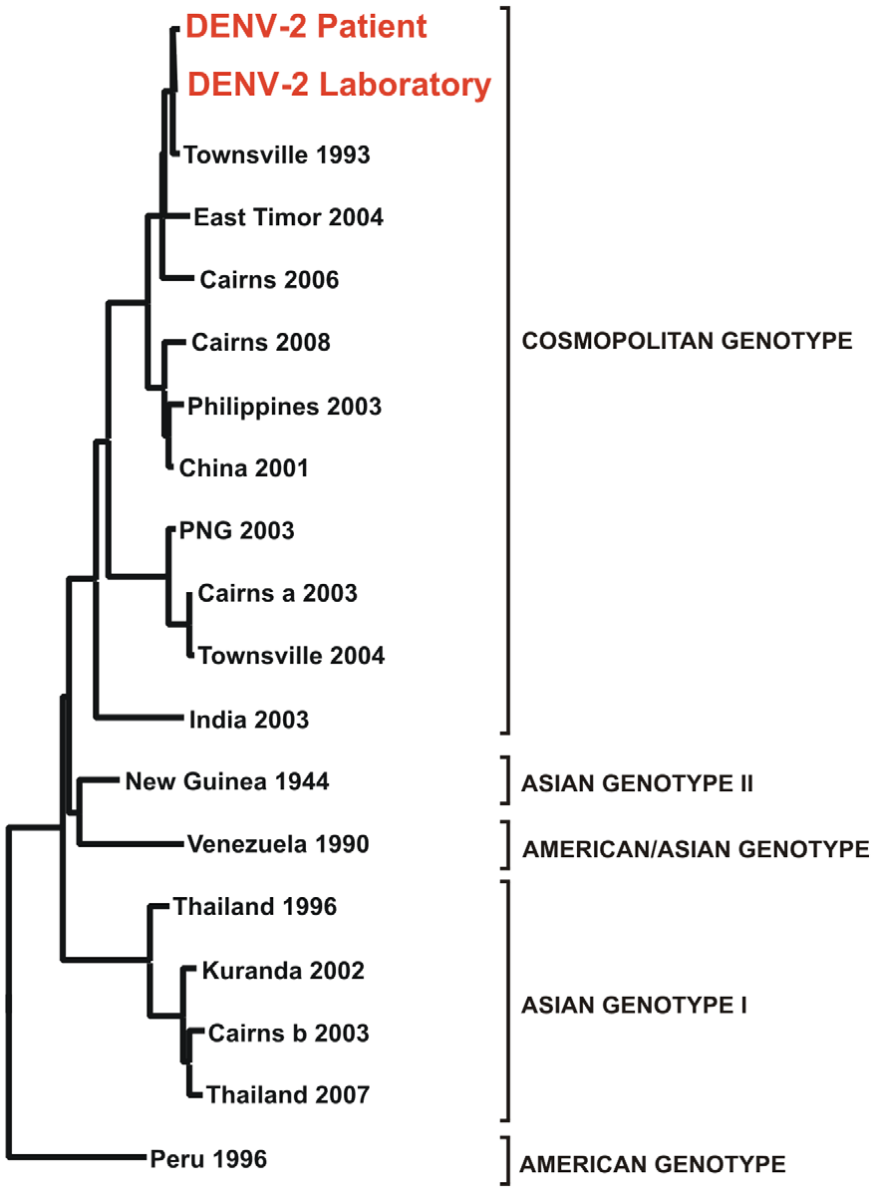
DENV-2 phylogenetic tree based on partial M and E gene nucleotide sequences depicting the relationship between the virus acquired by the patient and that used in laboratory experiments (highlighted). These are compared with other recent DENV-2 isolates such as Townsville 1993 that were imported or resulted in outbreaks in Queensland. Also shown are the genotypes of DENV-2 and representative strains from each grouping.

In support for this infection having been acquired in the laboratory, the antibody response was to the same virus serotype as was used during the laboratory experiment, and nucleotide sequencing analysis affirmed that the DENV-2 strain recovered from the patient was 99.8% homologous and therefore an identical strain to the virus that had been used (Figure 1). The DENV-2 strain used had been originally isolated during an outbreak in Townsville in 1993. After 3 days in hospital, the patient was discharged and within 48 hours all symptoms had resolved and the results of laboratory tests had returned to normal.

## Conclusions

There have been numerous reports of personnel acquiring incidental infections during manipulation of arboviruses within the laboratory [5], [8], [9]. However, to our knowledge, this is the first reported case where exposure during laboratory-based mosquito infection and transmission experiments has resulted in an acute DENV infection. In this instance, the experiments involved exposing colony-reared uninfected mosquitoes to an artificial blood meal containing DENV-2 via a membrane feeding apparatus. The high sequence homology and phylogenetic relatedness between the virus obtained from the patient and the virus used during the vector competence experiments confirms that they were identical strains and strongly suggests that the patient acquired the infection during the course of this procedure. Furthermore, these findings provide substantial evidence that the patient was not infected while travelling in Argentina, where DENVs do circulate. In any case, this is highly unlikely given the fact that the patient had returned from Argentina 4 weeks prior to developing dengue, an interval that greatly exceeds the normal incubation period for DENVs (between 3 and 14 days [10]). Finally, although north Queensland was experiencing concurrent outbreaks involving all four DENV serotypes at the time [11], the patient had not travelled to this region prior to developing dengue.

Upon notification of the case, the research facilities were independently inspected to assess the potential for further laboratory-acquired cases and the risk to the general public as well as to determine the potential route of virus exposure. Inspectors observed that the research facility and associated procedures adhered to the regulations as they apply to physical containment 2 (PC2) laboratories, the containment level required for experimentation with DENVs in Australia [6]. Importantly, it was concluded that the PC2 level of containment within the facilities significantly reduced the risk of exposure to other laboratory workers or the general public via experimentally infected mosquitoes. Furthermore, given that *Ae. aegypti* does not occur in southeast Queensland, the region where the patient resided, the risk of local transmission was considered to be negligible.

The investigations carried out in response to the case did not conclusively determine the specific route by which the patient was infected with DENV-2. It was noted that, although the majority of procedures involving live virus were performed in a class II biological safety cabinet (BSC), some aspects of the experiments were, by necessity, conducted on the bench outside the BSC. It was during this process that the patient may have been exposed to the virus within aerosolized blood droplets, resulting in mucocutaneous transmission, or perhaps may have become infected via contact through an unrecognized dermal abrasion. Indeed, mucocutaneous exposure was previously incriminated as the source of DENV infection in a health worker in the United States [12]. Alternatively, the route of exposure may have been via mosquito bite, as the patient reported being bitten by an unengorged mosquito that had escaped during the feeding period. Even if the mosquito had only probed the blood/virus mixture without feeding, or had even imbibed a small amount of the mixture, there would not have been sufficient time for the virus to replicate in the mosquito before transmission. Nonetheless, mechanical transmission of the virus via contaminated mosquito mouthparts cannot be completely excluded, as this phenomenon has been demonstrated previously with DENVs [13].

This case provides a timely reminder of the risk of arbovirus infection acquired by laboratory personnel through either vector or non-vector modes of transmission. It also highlights the importance of appropriate laboratory practices for containing infected mosquitoes and preventing contact with potentially infectious material, including the generation of potentially infectious aerosols. The use of personal protective equipment, including face mask and eye protection, and where possible, conducting all manipulations using live virus within a class II BSC, would be appropriate for prevention of laboratory-acquired arbovirus infections.

The patient has consented to publication of this case report.
